# ^99m^Tc-labelled PSMA ligand for radio-guided surgery in nodal metastatic prostate cancer: proof of principle

**DOI:** 10.1186/s13550-021-00762-1

**Published:** 2021-03-04

**Authors:** Michael Mix, Wolfgang Schultze-Seemann, Moritz von Büren, August Sigle, Mohamed A. Omrane, Markus T. Grabbert, Martin Werner, Christian Gratzke, Philipp T. Meyer, Cordula A. Jilg

**Affiliations:** 1 Department of Nuclear Medicine, Medical Center – University of Freiburg, Faculty of Medicine, University of Freiburg, Freiburg, Germany; 2 German Cancer Consortium (DKTK), Partner Site Freiburg, German Cancer Research Center (DKFZ), Freiburg, Germany; 3 Department of Urology, Medical Center – University of Freiburg, Faculty of Medicine, University of Freiburg, Freiburg, Germany; 4 Institute for Pathology, Medical Center – University of Freiburg, Faculty of Medicine, University of Freiburg, Freiburg, Germany

**Keywords:** Radio-guided surgery, PSMA, Prostate cancer, Salvage lymph node dissection, ^99m^tc-PSMA uptake in lymph nodes

## Abstract

**Purpose:**

Intraoperative identification of prostate cancer (PCa) lymph node (LN) metastases (LNM) detected by preoperative PSMA PET/CT may be facilitated by PSMA radio-guided surgery (RGS) with use of a γ-probe. Earlier we demonstrated excellent performance of the ^111^In-labelled PSMA ligand DKFZ-617 ([^111^In]In-PSMA-617) in RGS for ex situ distinction of LN vs LNM at lymphadenectomy (LA) at a single LN level. In comparison with indium-111, technetium-99m has better physical properties for γ-probe measurements, better availability and lower radiation exposure for patients and medical personnel. Against this background, we evaluated the uptake of ^99m^Tc-PSMA-I&S ligand at the level of single LN and its power to discriminate between unaffected LN and LNM.

**Methods:**

Six patients with PCa with the suspicion of LNM on preoperative PSMA-PET/CT underwent [^99m^Tc]Tc-PSMA-I&S RGS (4 salvage LA, 2 primary LA) with intravenous injection of [^99m^Tc]Tc-PSMA-I&S 24 h prior to surgery. Resected samples were isolated manually aiming at the level of single LN. Uptake measurements were done ex situ with a high-purity germanium detector. Receiver operating characteristic (ROC) analysis was performed based on [^99m^Tc]Tc-PSMA-I&S uptake expressed as lean body mass standard uptake value (SUL).

**Results:**

Separation of the tissue samples from 73 subregions resulted in 498 single samples. After final histopathology 356 LN, 160 LNM und 11 non-nodal PCa samples were identified. Median SUL of tumor-free samples (0.26) and samples with cancer (3.5) was significantly different (*p* < 0.0001). ROC analysis revealed an area under the curve (AUC) of 0.917 (95% CI 0.89–0.95). Using a SUL cutoff of 1.1, sensitivity, specificity, positive predictive value, and negative predictive values were 76.6%, 94.4%, 89.4% and 86.9%.

**Conclusion:**

Ex situ analysis of [^99m^Tc]Tc-PSMA-I&S uptake at single LN level showed good diagnostic performance for the ex situ distinction of tumor-bearing vs tumor-free LN during RGS.

## Introduction

Prostate cancer (PCa) is the most commonly diagnosed cancer in men [1, 2]. Pelvic and retroperitoneal lymph nodes (LN) are the first site for metastases [2, 3]. Although the impact of LA at radical prostatectomy has not been yet fully clarified, it remains the gold standard of staging PCa and an improvement of the oncological outcome could be suggested [4]. Regretfully, about 15–30% of the patients will develop a biochemical recurrence with elevated PSA level and clinical recurrence (metastases) possible at different anatomical sites [5, 6]. Regardless if LA is done at primary therapy or in case of recurrence, an accurate preoperative identification of LNM by, for instance, positron emission tomography/computed tomography (PET/CT) is a prerequisite for successful surgery [7, 8]. PET/CT targeting prostate-specific membrane antigen (PSMA) using ^68^Ga-labelled PSMA ligands has demonstrated an excellent ability to detect LNM prior to surgery and is widely used as a tool for staging before primary therapy and for restaging in the setting of biochemical relapse [7, 9–11]. Due to several advantages of the positron emitter fluorine-18, more and more frequently ^18^F-labelled PSMA ligands ([^18^F]F-PSMA-1007, [^18^F]F-DCFPyL) are used for PET/CT imaging [12–15].

If PET/CT indicates “regional pelvic LNM” as the only finding at clinical recurrence, surgical removal (i.e., “salvage lymphadenectomy”, salvage LA) [16, 17] of the lymphatic tissue or targeted radiotherapy may be suggested in patients in good general condition [7, 18, 19], but should be considered to be experimental and individual therapeutic approaches [1, 2]. Locating suspected LNM during surgery (e.g., salvage LA) is often very challenging in the case of small LNM and reduced accessibility to the LNM (e.g., because of atypical location of LNM and tissue adhesions). In order to address this issue, radio-guided surgery (RGS) using γ-emitting tracers had been introduced [20–24]. Suspected LNM could be identified during surgery using a γ-probe with acoustic feedback. Accordingly, the surgeon is able to conduct in situ and ex situ measurement of suspected regions and from resected tissue samples [25]. For [^111^In]In-PSMA-I&T RGS, the use of the γ-probe during salvage LA provided a specimen-based sensitivity of 83.6%, a specificity of 100%, and an accuracy of 93% [26]. Because of several well-known advantages (e.g., costs, availability, physical properties for the γ-probe detection, radiation exposure) PSMA tracers labelled with technetium-99m are currently preferred over those labelled with indium-111 [22, 27]. However, RGS is applied differently at the institutions [21, 28] and there are no data available about the exact uptake of [^99m^Tc]Tc-PSMA-I&S at a “single LN level” (manual separation of the resected tissue samples into single LN and LNM). By performing a precise ex-situ analysis with a high-purity germanium detector, we investigated the absolute tracer uptake and the performance of [^99m^Tc]Tc-PSMA-I&S in patients who underwent RGS and in whom we did a meticulous manual separation of the LN.

## Material and methods

### Patients

From 12/2017 to 03/2019, 38 patients with prostate cancer and the suspicion of exclusive LNM (without detectable bone or visceral metastases) on PSMA PET/CT underwent a LA after application of [^99m^Tc]Tc-PSMA-I&S prior to surgery. In a subset of 6/38 patients a single LN preparation and tracer uptake measurement of the samples was performed, being not a part of the clinical routine but addressing the issue of the current analysis. Two of these six patients underwent extended LA at radical prostatectomy for primary PCa. Four of six patients underwent a salvage LND on a compassionate-use basis because of biochemical recurrence (PSA > 0.2 ng/ml after radical prostatectomy) (Table 1). The LA in the six patients, regardless if at primary therapy or at the stage of biochemical recurrence was conducted to achieve a maximal tumor reduction. The large number of LN samples was also suitable for a dedicated analysis of tracer uptake, presented here. The sample processing (manual separation after surgery followed by direct measurement of the samples in a high-purity germanium detector) was planned and conducted in a prospective intention. The local ethics committee approved this data analysis (No. 562/15). Informed consent was obtained from each subject.

**Table 1:** Patient characteristics and outcome from 6 [^99m^Tc]Tc-PSMA-I&S RGS lymphadenectomies

No	Age at surgery (years)	PSA at surgery (ng/ml)	Gleason score at primary stage	Activity ^99m^Tc-PSMA (MBq)	LN/LNM removed overall (*n*)	LNM detected (*n*)	Removed non nodal** PCa-tissue (*n*)	Anatomical subregions at LA (*n*)	Manually separated “single-samples” (*n*)
Number of patients (extended lymph node dissections) overall *n* = 6									
1	69	2.27	4 + 3	680	79	15	–	18	78
2	49	128	5 + 4	660	135	42	1	12	84
3	63	67.63	4 + 4	692	123	8	–	15	126
4	68	0.99	4 + 4	537	36	2	–	8	56
5	65	11.78	4 + 4	674	50	8	–	12	58
6	52	54.82	4 + 3	584	93	85	10	8	96
Sum					516	160	11	73	498

Origin of tissue specimens from 73 subregions: *n*/73 (%)		
Right common iliac 7/73 (10%)	Left common iliac 6/73 (8%)	Aortic bifurcation 1/73 (1%)
Right external iliac 6/73 (8%)	Left external iliac 7/73 (10%)	Interaortocaval 3/73 (4%)
Right obturator iliac 4/73 (6%)	Left obturator iliac 4/73 (6%)	Paraaortal 4/73 (5%)
Right internal iliac 7/73 (10%)	Left internal iliac 5/73 (8%)	Miscellaneous* 9/73 (11%)
Right presacral region 6/73 (8%)	Left presacral region 4/73 (5%)	

*LNM* lymph node metastases, *LN* lymph node, *PSA* prostate specific antigen, *LA* lymphadenectomy, *PCa* prostate cancer

^*^miscellaneous regions: mesorectal, pillar of urinary bladder, region of the musculus piriformis, retroduodenal, renal hilum, ductus deferens

^**^non-nodal PCa-tissue: residuals of seminal vesicle (*n* = 7), and deferent duct (*n* = 3) and solid PCa-tissue in the of left fossa obturatoria (*n* = 1)

### ^68^Ga-PSMA-PET/CT and ^18^F-PSMA-PET/CT and imaging analysis

PSMA PET/CT was conducted with [^68^Ga]Ga-PSMA-11 and after availability of a ^18^F-labelled PSMA ligand at our department with [^18^F]F-PSMA-1007. For both tracers we used clinically established imaging protocols and uptake times as described by Jilg et al. [7] and Giesel et al. [15]. [^68^Ga]Ga-PSMA-PET/CT (*n* = 4) imaging was done 62 ± 2 min after injection of mean 220 ± 35 MBq and [^18^F]F-PSMA-PET/CT (*n* = 2) 124 ± 5 min after injection of mean 321 ± 26 MBq. A PSMA-positive lesion was defined as focal tracer accumulation greater than physiological local background activity. PET/CT study evaluation was done with co-registered PET and CT datasets using predefined PET window settings (inverted grey scale, SUV range: 0–5 g/ml for ^68^Ga-PSMA and 0–10 g/ml for ^18^F-PSMA). All patients showed increased focal ^68^Ga- or ^18^F-PSMA uptake in at least one pelvic and/or retroperitoneal region.

### [^99m^Tc]Tc-PSMA-I&S synthesis and application

Synthesis and radiolabeling of [^99m^Tc]Tc-PSMA-I&S was done as published by Robu et al. [29], i.v. tracer application for RGS was done 24 h prior to surgery (mean 638 ± 57 MBq). SPECT/CT was conducted a mean of 5–6 h after injection and 17–18 h prior to surgery to cross validate findings of PET/CT imaging, to serve as quality control for tracer application and distribution and to ensure sufficient tracer accumulation in the suspected PCa lesions. Two representative patient examples of PET and their corresponding SPECT images are shown in the Additional file 1: figure 1 and Additional file 2: figure 2.

### Lymphadenectomy

Open lymphadenectomy was performed as described by Jilg et al. [7]. The extent of LA was determined first by the aim to adhere to a template LA and according to the presence of PET-positive lesions: in the case of a pelvic PET-positive lesion(s), a bilateral pelvic LA was intended whenever possible. In the case of an additional PET-positive lesion in the retroperitoneum, a retroperitoneal LA was conducted. Subregions for a template pelvic bilateral LA were: common iliac vessels, external iliac vessels, obturator vessels, internal iliac vessels (presacral region). Subregions for a template retroperitoneal LA were: aortic bifurcation, aortal and caval region. Whenever permitted by the intraoperative circumstances (deviation from the template, e.g., caused by surgical difficulties) we adhered to this template. Nodal fibrofatty tissue (FFT) from each subregion was collected separately at surgery.

During LAs, γ-probe measurements were performed in situ and ex situ. γ-counts from ex situ measurements provided an assessment if the removed tissue specimen was presumably tumor-bearing or tumor-free. The number of counts from the γ-probe used intraoperatively (ex-situ measurement) gave the surgeon feedback if the suspected tumor tissue was resected or if the LA in this subregion had to be continued which ultimately increased the intensity of LA in this subregion. In cases in which ex situ γ-counts were very low (which indicates that tissue resected was free of tumor) and preoperative PET/CT predicts an LNM in the respective anatomical region, the surgeon extended the search for LNM. Results from γ-probe measurements during surgery were not evaluated in the current analysis because these measurements were done on unprocessed specimens, consisting of nodal FFT, LN and LNM.

### Sample processing

**Fig. 1:**
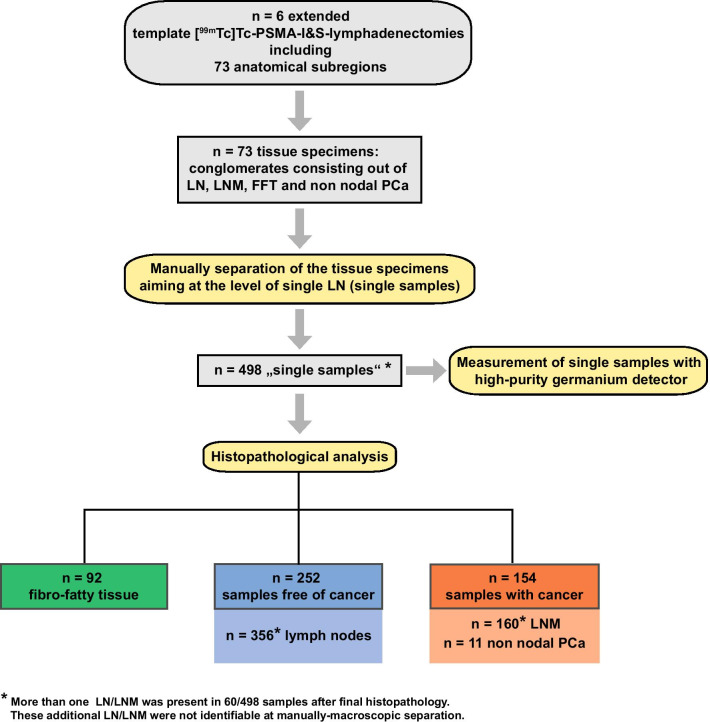
Workflow and tissue sample processing from 6 [^99m^Tc]Tc-PSMA RGS lymphadenectomies. Resected tissue specimens from subregions were manually separated to the level of “single samples” (aiming at single LN) that were measured with a high-purity germanium detector. After final histopathological analysis, 160 LNM and 11 non-nodal tumor samples were present in 154 manually separated single samples and 516 tumor-free lymph nodes were enumerated out of 252 tumor-free single samples

Following surgery, the specimens from all subregions were manually separated into single samples under guidance of an experienced pathologist (Fig. 1). Each single sample was weighed and numbered for further analysis. In 60/498 processed single samples, more than one LN and/or LNM was present after final histopathology. These additional LNs and LNMs were not identifiable during manually macroscopic separation. Samples with more than one LN or LNM were excluded in the further analysis.

### Analysis of trace uptake

Analysis of tracer uptake was performed analog as described by Mix et al. for indium-111 [23]. [^99m^Tc]Tc-PSMA-I&S radioactivity measurement of each single sample was done with a high-purity germanium detector (Canberra Inc., model GX2018-CP5+), calibrated with a multi-isotope reference source (type VZ-2139/NG3 from Eckert&Ziegler Nuclitec’s, DKD-accredited measurement laboratory in Germany) and cross calibrated for tissue sample geometry. Tracer uptake was calculated as percent injected dose per gram (%ID/g) and as SUL following PET standardized uptake value using lean body mass instead of body weight:

$${\text{SUL}} = \frac{{{\text{tissue}}\,{\text{sample}}\,{\text{activity}}\,({\text{Bq}})/{\text{tissue}}\,{\text{sample}}\,{\text{weight}}\,({\text{g}})}}{{\left( {{\text{injected}}\,{\text{activity}}\,({\text{Bq}}) \cdot {\text{e}}^{{ - {\text{ln}}\left( 2 \right) \cdot \Delta t/T_{1/2} }} } \right)/{\text{lean}}\,{\text{body}}\,{\text{mass}}\,({\text{g}})}},$$

with $\Delta t$ as delay between patient injection and time of surgery, *T*_1/2_ as half-life of technetium-99m. Lean body mass was calculated according to Janmahasatain et al. [30].

### Histopathological analysis

All resected LNs (i.e., the entire LN in the case of LNs ≤ 4 mm, one central slice in the case of LNs > 4 mm) were formalin-fixed and paraffin-embedded. The pathologist was not aware of the PET findings, the clinical estimate of the tissue from the surgeon or any of the tracer uptake measurements. Histopathologic evaluation was performed by one pathologist on hematoxylin and eosin (H&E) stained tissue slides.

### Statistics

Descriptive statistics were obtained by calculating mean, standard deviation (SD), median and range. Continuous variables were compared with a two-sided unpaired Mann–Whitney *U* test. A receiver operating characteristic (ROC) analysis was performed in order to analyse the diagnostic performance of [^99m^Tc]Tc-PSMA-I&S tracer uptake for identification of LNM. Sensitivity, specificity, positive predictive value (PPV) and negative predictive value (NPV) were analyzed at the most appropriate cutoff value established as the one with the highest result of the sum of sensitivity and specificity (Youden index). The 11 tumor samples with non-nodal PCa were handled as LNM in statistical analysis. Prism8 GraphPad was used for statistical calculations.

## Results

### Lymphadenectomy

Clinical patient characteristics such as age and Gleason-score and the outcomes from LAs of the 6 patients are summarized in Table 1. The 6 patients were part of a larger cohort that underwent RGS because of suspected LNM in a PSMA-PET/CT and had a high-risk PCa stage . Time from PSMA PET/CT to LA was 2 ± 1.5 months. Median PSA at surgery was 33.3 ng/ml (range 0.99–127 ng/ml) (Table 1). Figure 1 shows the workflow of the sample processing. Even though only 6 patients underwent surgery a relatively large number of 516 LN from 73 subregions had been removed (mean 86 ± 39 per patient). Median 11.5 (mean 26.7 ± 31.9) LNM were identified per patient. From each subregion, median 7 (mean 6.6 ± 1.7) single samples were identified. The majority of the samples and the achieved single samples originated from pelvic regions (Table 1).

### Histopathology

Histopathological analyses of the 498 single samples yielded 92 FFT samples, 154 samples with cancer (160 LNM, 11 non-nodal tissue), and 252 tumor-free samples (indeed 356 tumor-free LN). The 11 samples with non-nodal PCa comprised out of residuals of seminal vesicle (*n* = 7), deferent duct (*n* = 3) and solid PCa-tissue in the of left fossa obturatoria (*n* = 1). In 60/498 single samples more than one LN (macroscopically inseparable) was present at final histopathology, resulting in 516 LN overall (Table 1).

### Tracer uptake

**Fig. 2:**
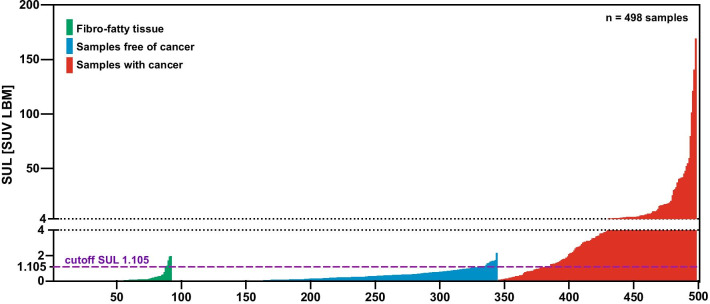
Alignment of the SUL of tumor-containing and tumor-free samples illustrating the diagnostic performance of [^99m^Tc]Tc-PSMA-I&T for discrimination between samples with and without cancer cells

**Fig. 3:**
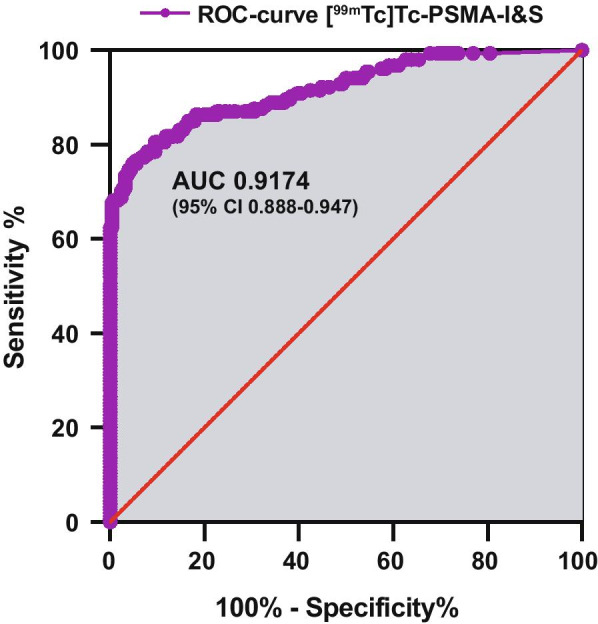
ROC analyses revealed an AUC of 0.9174 (95% CI 0.89–0.95) for [^99m^Tc]Tc-PSMA-I&T (154 samples with tumor, 252 samples without tumor)

Data on weight, tracer uptake (SUL) and percent of injected dose per gram (%ID/g) from single samples removed at [^99m^Tc]Tc-PSMA-I&S RGS are shown in Table 2. Tumor-free samples and tumor-bearing samples showed clearly significantly different values (*p* < 0.0001 each) for all 3 parameters (weight, tracer uptake, SUL). There was no correlation between tissue weight and tracer uptake or SUL in the tumor-free and tumor-bearing samples. Tracer uptake of the lightest LNM and LN (both 0.01 g) was 1.8 × 10^–4^ and 1.0 × 10^–6^%ID/g while the heaviest LNM (6.0 g) and LN (7.8 g) showed a tracer uptake of 6.9 × 10^–5^ and 1.5 × 10^–6^%ID/g. The weight of the LNM and LN with the lowest uptake (1.9 × 10^–8^ and 1.1 × 10^–9^%ID/g) was 0.05 and 1.3 g while the weight of the LNM and LN with the highest uptake (2.9 × 10^–3^ and 8.8 × 10^–5^%ID/g) was 0.03 and 0.05 g. The SUL values for all 498 single samples from [^99m^Tc]Tc-PSMA-I&S RGS are shown aligned for the three different tissues in Fig. 2. A receiver operating characteristic (ROC) analysis revealed an area under the curve (AUC) of 0.9174 (95% CI 0.89–0.95) for [^99m^Tc]Tc-PSMA-I&T RGS (154 samples with tumor, 252 samples without tumor). The ROC curve is shown in Fig. 3.

**Table 2:** Single sample measurements from [^99m^Tc]Tc-PSMA-I&S RGS

	Weight (g)		Tracer uptake in %ID/g		SUL (SUV LBM)	
FFT, *n* = 92						
Mean	0.62		0.00022		0.16	
± SD	± 0.96		± 0.00064		± 0.37	
Median	0.16		0.0		0.010	
Range	0.01–4.2		0.0–0.003		0.0–2.0	

		*p* value Mann–Whitney *U* test		*p* value Mann–Whitney *U* test		*p* value Mann–Whitney *U* test
LN samples*, *n* = 252						
Mean	0.63		0.00059		0.37	
± SD	± 1.1		± 0.00075		± 0.40	
Median	0.18		0.0		0.26	
Range	0.01–7.8		0.0–0.0040		0.0–2.2	
LNM samples*, *n* = 154		< 0.0001 (LN vs LNM)		< 0.0001 (LN vs LNM)		< 0.0001 (LN vs LNM)
Mean	0.84		0.019		12.0	
± SD	± 1.2		± 0.041		± 24.0	
Median	0.36		0.0050		3.5	
Range	0.01–6.0		0.0–0.29		0.0–169	

*LN* lymph node, *LNM* lymph node metastases, *FFT *fibro-fatty-tissue, *PCa *prostate cancer

^*^60/498 LN-samples containing more than one LN/LNM resulting in overall 356 LN and 171 LNM (including 11 non-nodal PCa tissue)

### Power of discrimination of [^99m^Tc]Tc-PSMA-I&S in single lymph nodes

To describe the ability of discrimination of tumor-bearing versus tumor-free tissue, we focused on 406 single samples (excluding FFT samples) (Table 3). Using an SUL cutoff of 1.105, sensitivity, specificity, positive predictive value and negative predictive value was 76.6% (118/154), 94.4% (239/252), 89.4% (118/132), and 86.9% (238/274) (Table 3).

**Table 3:** Accuracy of tumor discrimination of ^99m^Tc-PSMA ligand using SUL, cutoff: 1.105 (Youden-index)

	Sensitivity %	Specificity %	PPV %	NPV %
*n* = 406 samples	76.6 (118/154) CI (0.69–0.83)	94.4 (238/252) CI (0.91–0.97)	89.4 (118/132) CI (0.83–0.94)	86.9 (238/274) CI (0.82–0.91)
*n* = 154 samples with tumor				
*n* = 252 samples free of cancer				

*PPV/NPV* positive/negative predictive value

## Discussion

Conditions for optimal RGS (tracers, protocols) are still in development and under investigation [27]. Available reports on the application of ^99m^Tc-labelled PSMA ligands for detection of LNM during RGS were based on the analysis of “sample-mixtures” from one anatomical region consisting out of LN, LNM and FFT [22, 27]. Consequently, tracer activity uptake measured in such samples represents the sum of tracer distribution in different kind of tissues with different uptake characteristics (LN, LNM, FFT). Just as well, results from intraoperative gamma probe measurements (regardless if in situ or ex situ) might be blurred due to, e.g., multiple surrounding healthy tissue or unspecific tracer uptake in, e.g., healthy lymph nodes or other tissue. Accordingly, the exact performance of the ^99m^Tc-labelled PSMA ligand and its diagnostic accuracy (performance) on a single LN basis could not be evaluated.

To our best knowledge, the present analysis is the first clinical investigation of tracer uptake for [^99m^Tc]Tc-PSMA-I&S RGS in tumor tissue at the level of single lymph nodes. The large number of LN and LNM samples removed at surgery (406 in 6 patients) indicates that larger tumor formations were not missed. It suggests a comprehensive data record to evaluate the performance of the tracer for the distinction between tumor-bearing and tumor-free LN. SUL was significantly different between samples with PCa and non-affected samples (Table 2 and Fig. 2). To reach the mean tracer uptake in tumor containing LN it would take more than 30-fold of the amount of mean tracer uptake in tumor-free LN (Table 2). This contrast is 2.5-fold lower than we found for [^111^In]In-PSMA-617 [23]. The reduced contrast for ^99m^Tc]Tc-PSMA-I&S is caused by a 34% higher average tracer uptake in unaffected LN and a 66% lower average tracer uptake in LNM. A reason for this could be the different treatment protocol. While RGS was done with a sufficient count rate for ^111^In-labelled PSMA ligands (2.8 days half-life) 48 h after injection of the tracer, RGS with ^99m^Tc-labeled PSMA ligands (6 h half-life) must be done earlier, in our study 24 h after tracer injection. Nevertheless, both tracers demonstrate the power to distinct between unaffected LN and LNM and therefore for the use for RGS. Another finding was a relatively high variation of the tracer uptake in the samples and that the range of uptake between LN and LNM overlap. Using the Youden Index as SUL cutoff, more false-positive (23.4% vs 7.9%) and false-negative (10.6% vs 5.4%) single samples were observed with technetium-99m than with the indium-111. All results based on ex situ measurements under optimal conditions like a high sensitivity germanium detector and separated single lymph nodes without any disturbing background signal from other tissue. Measurements of mixed tissue samples (LNM, LN lymph nodes with fibro fatty tissue) with a γ-probe during surgery, especially in situ, may be still challenging due to additional unavoidable physiological and unspecific background signal.

An important study from Maurer et al. [22] showed for ^99m^Tc labelled PSMA RGS in 31 patients undergoing salvage LND a specimen-based sensitivity of 83.6%, a specificity of 100%, a positive predictive value of 100% and a negative predictive value of 89.2%. These results are apparently better than the data in this study but they are hardly to compare because of the different surgical approaches. Based on PET/CT findings, Maurer et al. performed primarily a unilateral targeted RGS-lymphadenectomy with subsequent intraoperative γ-probe measurements to exclude additional lesions. Our approach was to adhere whenever possible to a bilateral template lymphadenectomy, both with subsequent intraoperative γ-probe measurements for verification. The latter approach might be less vulnerable to miss false negative LNM or very small LNM missed by PET/CT or, e.g., by a weak γ-probe signal due to a low [^99m^Tc]Tc-PSMA-I&S accumulation. Furthermore, the object of this study was to analyse a high number of single lymph nodes (prepared out of 498 single samples from 6 patients in this study) but not mixed tissue samples (136 specimens from 31 patients by Maurer et al. [22]). Additional optimization of the well-known widespread pencil γ-probes for an intraoperative situs or for robotic surgery might improve the approach. To address this issue Oosterom et al. developed a first DROP-IN γ-probe with a scanning direction range between 0° and 180° [31]. It can be assumed that the trend towards assisted respective guided surgery such as RGS will potentially improve the results of salvage LA.

## Limitations

The main limitation of this study is the small number of patients and the known inhomogeneity of PCa (e.g., Gleason score, PSA at surgery) which makes a comparison to the results with other traces like ^111^In-labeled PSMA ligands [23] difficult. Even if there is a high number of single LN and LNM samples (*n* = 406), in terms of tumor progression (number of LNM), the six patients are also very inhomogeneous. For example, patient no. 6 revealed a dramatically high number of LNM overall (*n* = 85) and showed a high PSA value, even after surgery (Additional file 3: table 1).

Generally, there may be an additional bias because only patients with suspected LNM on a PSMA-PET/CT and therefore known PSMA-positive lesions were included in this study.

Finally, it remains to be examined how the application can be optimized for ex-situ and in-situ measurements of sample mixtures with γ-probes and how dual labelled PSMA-targeting agents for radio- and fluorescence guides surgery can improve this approach [27].

## Conclusion

Ex situ analysis of [^99m^Tc]Tc-PSMA-I&S uptake at single LN level showed good performance for ex situ distinction of tumor-bearing vs tumor-free LN during RGS.

## Supplementary Information

**Additional file 1. Figure 1**: Preoperative [^69^Ga]Ga-PSMA-PET/CT (A) and corresponding [^99m^Tc]Tc-PSMA-SPECT (B) for verification of tracer injection and accumulation from patient No 2. A_1_/B_1_: maximum intensity projection, A_2_/B_2_: transaxial, A_3_/B_3_: sagittal, A_4_/B_4_ coronal slices and A_5_: fused transaxial PET/CT.

**Additional file 2. Figure 2**: Preoperative [^18^F]F-PSMA-PET/CT (A) and corresponding [^99m^Tc]Tc-PSMA-SPECT (B) for verification of tracer injection and accumulation from patient No 4. A_1_/B_1_: maximum intensity projection, A_2_/B_2_: transaxial, A_3_/B_3_: sagittal, A_4_/B_4_ coronal slices and A_5_: fused transaxial PET/CT.

**Additional file 3:** Clinical follow up of the 6 patients.

## Data Availability

The datasets used and analyzed in this study are available from the corresponding author on reasonable request.

## Abbreviations

%ID/g: Percent injected activity per gram
ADT: Androgen deprivation therapy
AUC: Area under the curve
CT: Computer tomography
FFT: Fibrofatty tissue
LA: Lymphadenectomy
LN: Lymph node
LND: Lymph node dissection
LNM: Lymph node metastasis
NPV: Negative predictive value
PET: Positron emission tomography
PPV: Positive predictive value
PCa: Prostate cancer
PSA: Prostate-specific antigen
PSMA: Prostate-specific membrane antigen
RGS: Radio-guided surgery
ROC: Receiver operating characteristic
SPECT: Single photon emission computer tomography
SUV: Standard uptake value
SUL: Standard uptake value normalized to lean body mass instead of body weight
