# Supplementary figures and images for: 99mTc-labelled PSMA ligand for radio-guided surgery in nodal metastatic prostate cancer: proof of principle
Source: EJNMMI Res. 2021 Mar 4;11:22. doi: 10.1186/s13550-021-00762-1 (PMC7933311; doi:10.1186/s13550-021-00762-1)

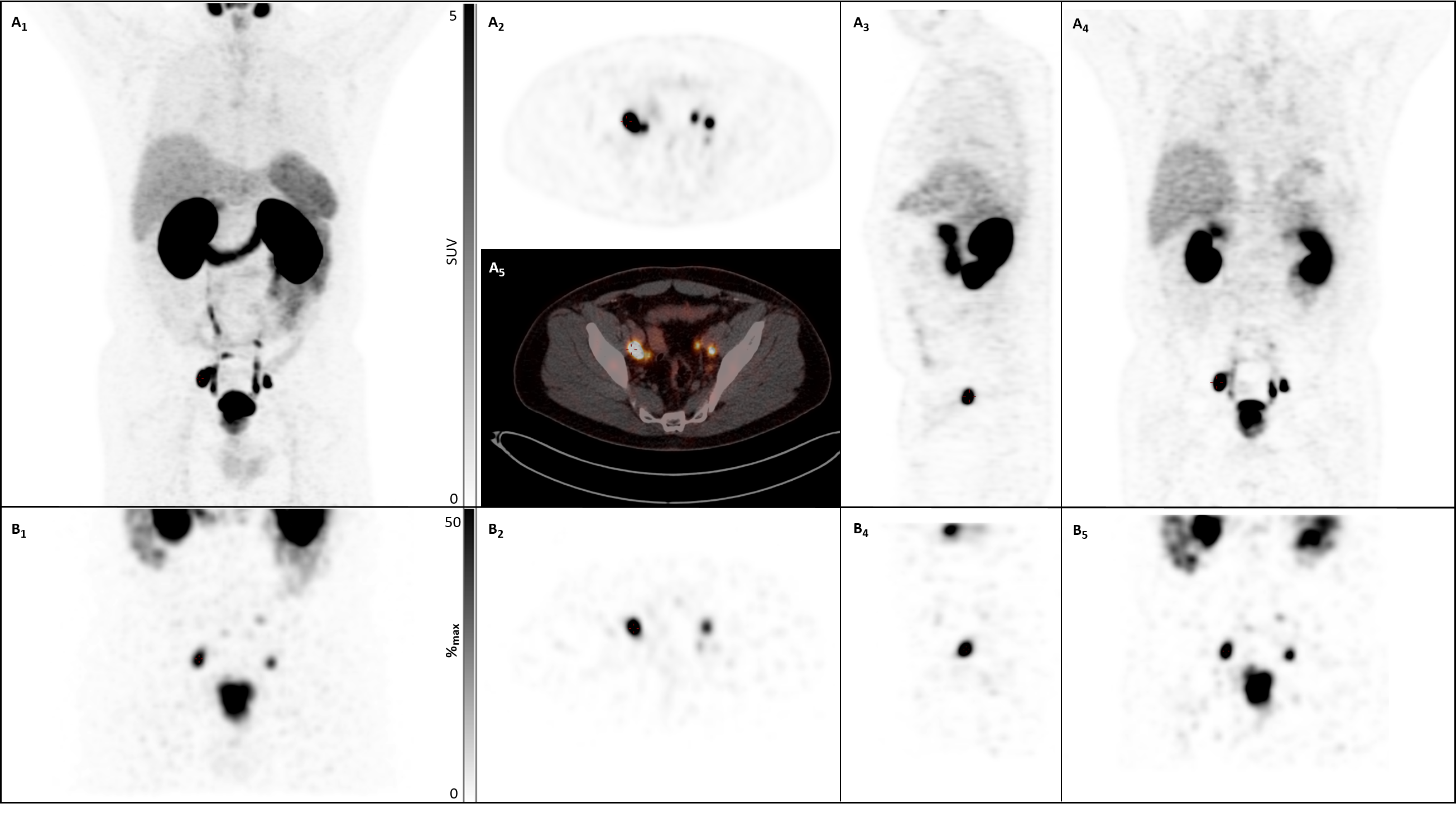

Supplement: Supplementary file 1 — Additional file 1. Figure 1: Preoperative [69Ga]Ga-PSMA-PET/CT (A) and corresponding [99mTc]Tc-PSMA-SPECT (B) for verification of tracer injection and accumulation from patient No 2. A1/B1: maximum intensity projection, A2/B2: transaxial, A3/B3: sagittal, A4/B4 coronal slices and A5: fused transaxial PET/CT. [file 13550_2021_762_MOESM1_ESM.tif]

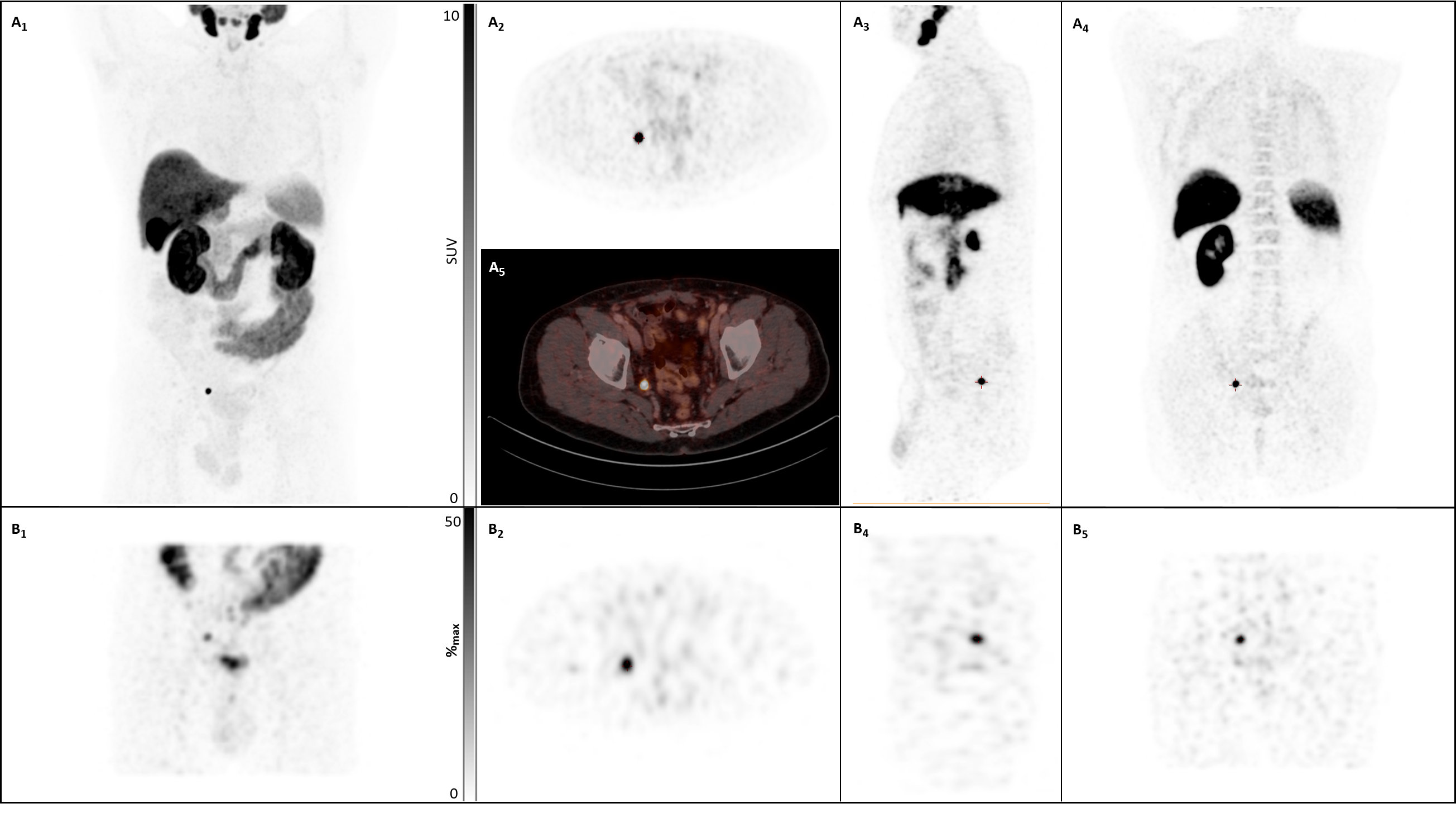

Supplement: Supplementary file 2 — Additional file 2. Figure 2: Preoperative [18F]F-PSMA-PET/CT (A) and corresponding [99mTc]Tc-PSMA-SPECT (B) for verification of tracer injection and accumulation from patient No 4. A1/B1: maximum intensity projection, A2/B2: transaxial, A3/B3: sagittal, A4/B4 coronal slices and A5: fused transaxial PET/CT. [file 13550_2021_762_MOESM2_ESM.tif]
