# Supplementary material for: 99mTc-labelled PSMA ligand for radio-guided surgery in nodal metastatic prostate cancer: proof of principle
Source: EJNMMI Res. 2021 Mar 4;11:22. doi: 10.1186/s13550-021-00762-1 (PMC7933311; doi:10.1186/s13550-021-00762-1)
Supplement: Supplementary file 3 — Additional file 3: Clinical follow up of the 6 patients. [file 13550_2021_762_MOESM3_ESM.docx]

**Supplementary table 1**

Clinical follow up

| **Patient N°** | **Latest follow up (months) *** | **PSA at latest follow up (ng/ml)** | **Radiotherapy conducted after RGS (Yes / No)** | **Clinical Stage at latest follow up** |
| --- | --- | --- | --- | --- |
| **1** | 32,9 | 0,15 | yes, (pelvic lymphatic region) | No ADT, stable disease |
| **2** | 20,3 | 17,1 | yes, (pelvic lymphatic region) | ADT with Enzalutamide because of progress |
| **3** | 1,5 | 2,6 | yes, (pelvic lymphatic region) | No ADT, stable disease |
| **4** | 22,7 | 0,29 | yes, (pelvic lymphatic region) | No ADT, stable disease |
| **5** | 18,5 | 0,008 | yes, (pelvic lymphatic region) | ADT with Profact, stable disease |
| **6** | 17,0 | 90,2 | yes, (pelvic lymphatic region) | Lutetium-177-PSMA-therapy, PC-progress |

PSA = Prostate Specific Antigen, ADT androgen deprivation therapy

*latest follow up calculated from surgery
